# A megawatt-level surface wave oscillator in Y-band with large oversized structure driven by annular relativistic electron beam

**DOI:** 10.1038/s41598-018-25466-w

**Published:** 2018-05-03

**Authors:** Jianguo Wang, Guangqiang Wang, Dongyang Wang, Shuang Li, Peng Zeng

**Affiliations:** 1 Science and Technology on High Power Microwave Laboratory, Northwest Institute of Nuclear Technology, P. O. Box 69-1, Xi’an, 710024 China; 2 Key Laboratory for Physical Electronics and Devices of the Ministry of Education, Xi’an Jiaotong University, Xi’an, 710049 China

## Abstract

High power vacuum electronic devices of millimeter wave to terahertz regime are attracting extensive interests due to their potential applications in science and technologies. In this paper, the design and experimental results of a powerful compact oversized surface wave oscillator (SWO) in Y-band are presented. The cylindrical slow wave structure (SWS) with rectangular corrugations and large diameter about 6.8 times the radiation wavelength is proposed to support the surface wave interacting with annular relativistic electron beam. By choosing appropriate beam parameters, the beam-wave interaction takes place near the π-point of TM_01_ mode dispersion curve, giving high coupling impedance and temporal growth rate compared with higher TM_0*n*_ modes. The fundamental mode operation of the device is verified by the particle-in-cell (PIC) simulation results, which also indicate its capability of tens of megawatts power output in the Y-band. Finally, a compact experimental setup is completed to validate our design. Measurement results show that a terahertz pulse with frequency in the range of 0.319–0.349 THz, duration of about 2 ns and radiation power of about 2.1 MW has been generated.

## Introduction

Numerous applications, such as plasma diagnostic in nuclear fusion, high data rate communications, remote high-resolution imaging, chemical spectroscopy, materials research, deep space research and communications, basic biological spectroscopy and biomedical diagnostics, are stimulating great interest in high power vacuum electronic devices (VEDs) of millimeter wave to terahertz regime^1–5^. Basically, the VEDs are classified into two types, one is the slow wave device, and the other is the fast wave device. The gyrotron device, a typical fast wave device with the phase velocity of the electromagnetic wave greater than the speed of light, has been developed in the terahertz range from 0.2–1.3 THz^6–8^. Glyavin *et al*.^6^ studied a pulsed gyrotron driven by the electron beam with voltage 20–25 kV and beam current up to 5 A^6^. At a 38.5 T magnetic field, the gyrotron generated coherent radiation power of 1.5 kW at 1.022 THz frequency in 50 μs pulses. Glyavin *et al*.^7^ developed a pulsed gyrotron driven by a 70 kV, 15 A electron beam, the beam power is on the order of 1 MW at the frequency of 0.67 THz with the duration of 10–20 ms^7^. In the Y band, Bandurkin *et al*. designed a continuous wave large-orbit gyrotron, under the condition 30-keV/0.7 A/5 T, it was aimed to generate hundreds of watts at the third cyclotron harmonics at the frequencies 0.39 THz^8^.

Compared with the fast wave devices, which require the large-scale particle accelerators and/or complex intense magnetic systems^6–9^, the slow wave devices are considered promising generators for their simpler structures and much lower magnetic field requirements. However, their structural dimensions scale down rapidly as the frequency goes up, causing some key problems to be solved, such as the limitation of power capacity, internal breakdown, and difficulties of fabrications and configurations of the device. One of the solutions is to utilize the planar structure compatible with modern micromachining technologies^10–14^, but the power capacity and the beam area are still small for device with pencil-like beam, while currently the generation and transport of sheet beam with small dimensions are challenging, especially for the high power applications^15^. Another solution is to introduce the oversized slow wave structure (SWS) in the cylindrical device, whose transverse diameter *D*_0_ is several times the radiation wavelength *λ*_0_, to increase the power-handling capability and beam surface area while reducing the fabrication difficulties moderately^16,17^.

Based on the approach of the oversized SWS, there are mainly two kinds of high power devices investigated widely in the terahertz range. The first one is the superradiant Cherenkov device, which is originated from the superradiance effect in optics^18^, and the coherent emission from the entire volume of the active medium occurs due to the development of microbunching and slippage of the wave with respect to electrons caused by the difference between the group velocity of the electromagnetic wave and the translational velocities of the particles^19^. Yalandin *et al*. studied experimentally the superradiant pulse with the frequency of 150 GHz by using the 180 keV electron beam, the measured peak power was 5–10 MW with the duration less than 1 ns and the rise time less than 75 ps^20^. Zhang *et al*. studied the Cherenkov superradiance with high peak power by using the particle-in-cell (PIC) simulations^21^, and presented the effects of the structural parameters, periodic number of SWS, and the driving parameters of the electron beam (driving voltage, current and its duration) on the output performance of the generated terahertz wave^22^. Ginzburg *et al*. studied the generation of subterahertz superradiant pulses based on excitation of a surface wave from relativistic electron bunches by using the quasioptical approach and PIC simulations, and obtained experimentally the subterahertz SR pulses with a central frequency of 0.14 THz, a ultrashort duration of 150 ps, and the high peak power of 50–70 MW^19^. Though very high powers were obtained by the SR mechanism, the durations of the terahertz pulse were very narrow, often sub-nanoseconds.

The second approach for generating high power terahertz wave is the oversized surface wave oscillator (SWO). By applying the quasioptical theory, Ginzburg *et al*. studied the planar SWO driving by the sheet electron beam^23^, and the cylindrical SWOs with one- and two-dimensional SWSs excited by the annular electron beams^19,24,25^. In China, our research group carried out extensive researches on the oversized SWOs driven by the annular relativistic electron beams^26–32^, experimental results indicated that a compact relativistic SWO generated 154 GHz pulse with repetition rate of 10 and power of 2.6 MW^27^, and its succedent improved version could radiate a single pulse with frequency of 149 GHz and power of 5 MW^28^. In Japan, Gong *et al*. developed cylindrical SWOs with operation frequencies in the range of 166–173 GHz and 182–200 GHz and radiation powers on the order of killowatts by using annular electron beams less than 100 kV^33^. In South Korea, Min *et al*. designed the 0.1–0.5 THz oversized BWO by using 500 kV–5 kA electron beam^34^. To pursue high electron current and small circuit thermal deposition, even CPI Inc. has been considering adopting this kind of configuration to inprove the performance of high frequency extended interaction klystron (EIK)^10^. Therefore, the cylindrical slow wave devices with oversized structures are competitive to generate the high power terahertz waves.

Currently, most experiments on the high power slow wave devices in the terahertz range are conducted in the frequency range below 200 GHz, above which many research works are conducting, but most of them are still at the stages of theoretical designs and numerical simulations^34^. In this work, a compact oversized SWO driven by the annular relativistic electron beam is proposed and experimented with operation frequency in the Y-band.

## Physical Design and Analysis

**Figure 1:**
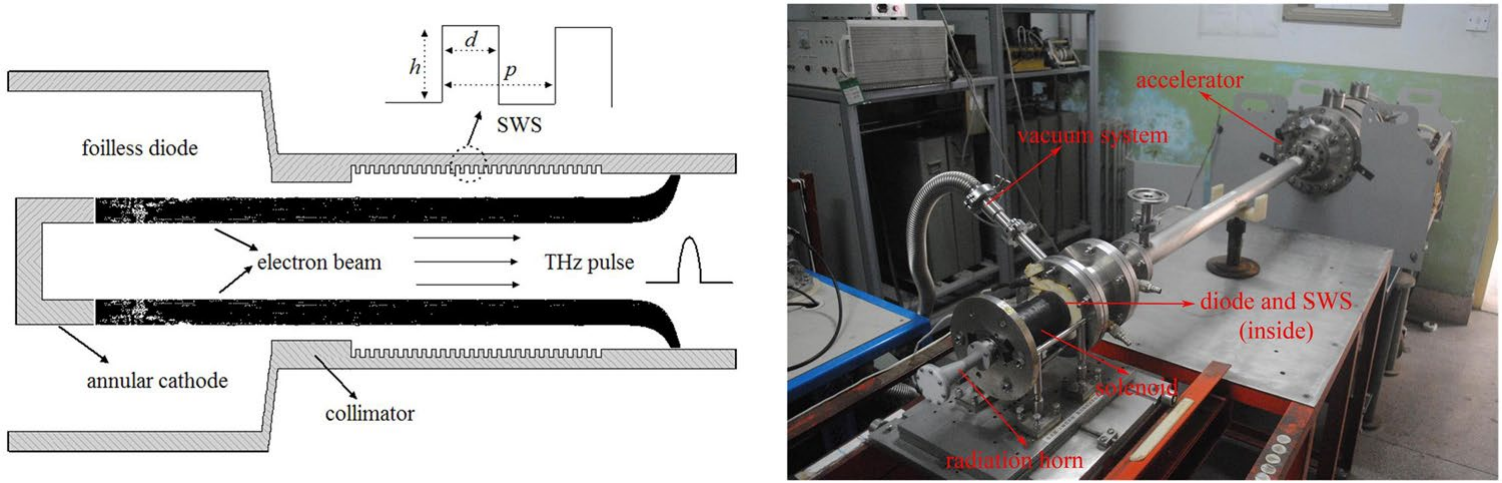
Schematic and apparatus of the oversized Y-band SWO.

As a typical Cerenkov oscillator, the oversized SWO utilizes an intense electron beam interacting with surface wave excited in the oversized SWS to obtain highly efficient output and mitigate the fabrication difficulty^25,35^. It is worth mentioning that harmonics of the operation mode are all surface waves in this device. Referring to the subterahertz SWO in our previous work^27^, the relativistic SWO in Y-band is designed for the electron beam with energy ranging from 300–500 keV, which can be provided by a compact accelerator in our laboratory^28^, and shown in Fig. 1 (the tube is immersed in an axially confined magnetic field). As usual, the foilless diode with annular cathode is used to emit required intense electron beam. The profile of SWS is rectangularly corrugated for easy fabrication and high coupling impedance^22^. Its inner radius is set to be 3 mm as a trade-off between the fabrication cost-effectiveness and mode control. A collimator is mounted before the SWS as an electron beam limiter to intercept the electrons from the beam edge which probably bombards the SWS wall.

**Figure 2:**
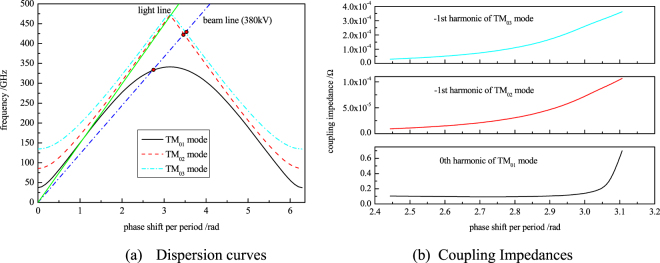
Dispersion curves and coupling impedances of TM_01_, TM_02_ and TM_03_ modes.

To support the surface wave interacting with given relativistic electron beam, the structural parameters are designed as follows: *p* = 0.32 mm, *h* = 0.12 mm, *d* = 0.18 mm. The small-signal linear theory is used to calculate the dispersion lines for the cold cavity and growth rate for the hot cavity. Here some assumptions are made: (1) infinitely long SWS; (2) infinitely strong magnetic field; (3) only axial movement of the electron beam; and (4) infinitely thin electron beam. Figure 2 illustrates the calculated dispersion curves and coupling impedances of the three lowest TM_0*n*_ modes^36^, where the Doppler lines of light and electron beam with voltage of 380 kV are also depicted. Obviously, the beam line intersects TM_01_ mode curve near π point in the 0*th* harmonic zone, while the interaction points with other two modes are both in the −1st harmonic region. All these intersections are below the light line, indicating that the corresponding harmonics are the surface wave. However, the 0*th* harmonics of TM_02_ and TM_03_ modes are above the light line and accordingly the volume waves. Since the 0*th* harmonic dominates the slow wave, only TM_01_ mode is surface wave in this oversized SWS, and its maximum longitudinal electric field appears near the inner wall.

As the oversized ratio of the SWS is as high as 6.8, the mode selection of TM_01_ mode become critical in the design of the device^29–31^. According to the unique electric field distribution of TM_01_ mode, we set the distance between the electron beam and SWS wall 0.3 mm to fully interact with the surface wave. Here the decreasing scale of the surface wave toward the center and the difficulty to implement this configuration in the experiments are both considered. The coupling impedances of the spatial harmonics that synchronize with electron beam for TM_01_, TM_02_ and TM_03_ modes are calculated and shown in Fig. 2(b). Enormous differences in the several orders of magnitude are found between the results of TM_01_ mode and other modes. Moreover, the differences become larger accompanying with beam-wave interaction point of TM_01_ mode moving towards π point on the dispersion curve. Therefore, only TM_01_ mode can be excited and amplified in the oversized SWS for the relativistic electron beam at selected radial position, and the fundamental mode operation of the proposed Y-band SWO is theoretically achieved.

## Pic Simulations

**Figure 3:**
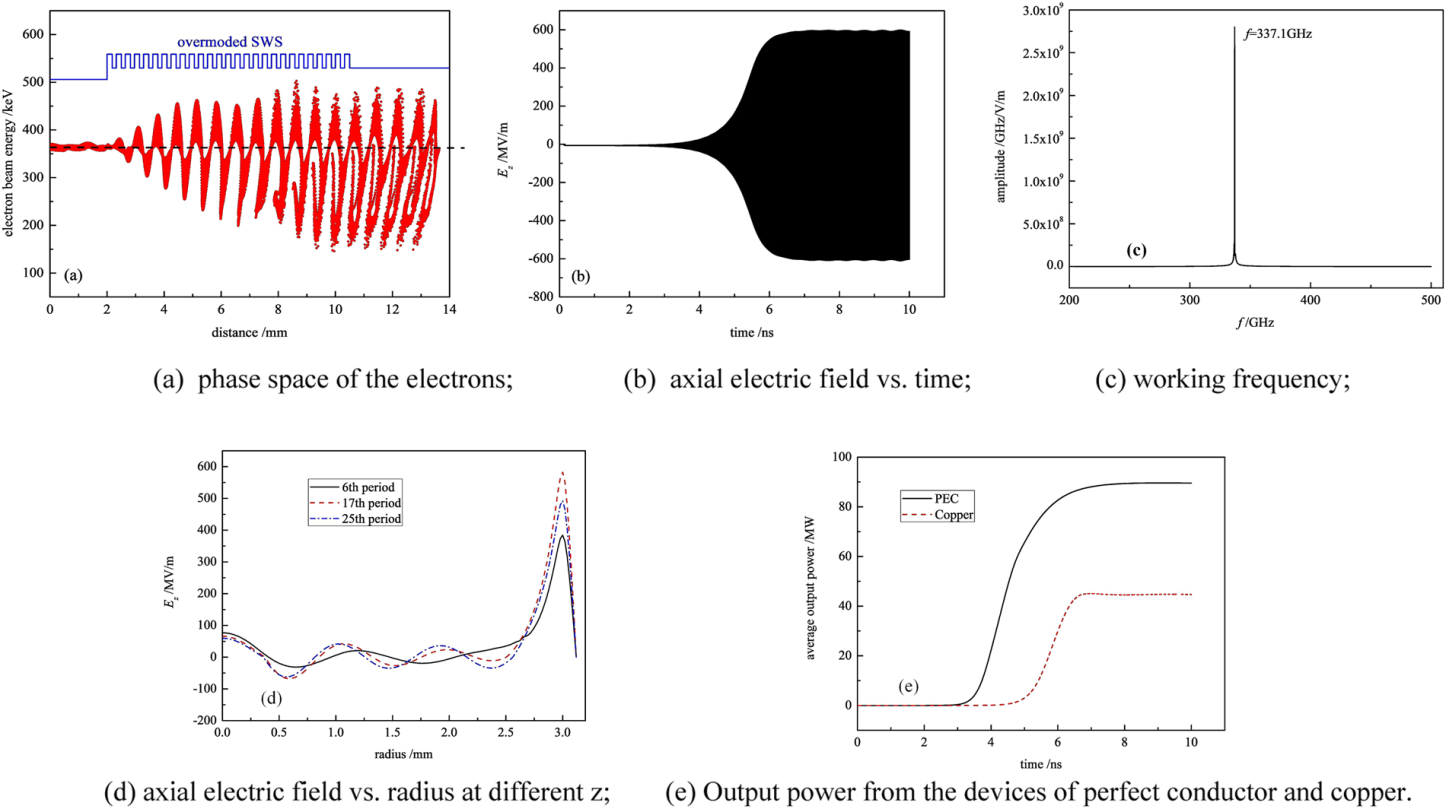
PIC simulation results.

To optimize other structural parameters of the proposed SWO and figure out its operation mode, the simulations are performed by using a fully electromagnetic PIC code UNIPIC-3D^37^, in which the relativistic Newton-Lorentz force equation and Maxwell’s equations are solved on conformal meshes, and the distribution loss on the device wall is included^38,39^. After dozens of computation cycles, the main dimensions of the structure of our device are obtained as follows: thickness of electron beam 0.3 mm, radius of collimator 2.9 mm, inner radius of SWS 3 mm, and period number of SWS 27. The optimized period number of the SWS here matches the beam voltage to ensure that only one discrete axial TM_01_ mode oscillates^25^. The typical PIC simulation results are shown in Fig. 3. The voltage and current of the electron beam generated by foilless diode are 380 kV and 2.2 kA, respectively, and the metal material of the device is copper. It is clearly indicated from Fig. 3(a) that the electron beam gets strong modulation in the SWS, and its modulation current could reach almost 9 kA accoding to further simulation. Figure 3(b,c) show the axial electric field *E*_*z*_ at certain position in the SWS and its corresponding frequency spectrum, respectively. High electric field is successfully excited at frequency of 337.1 GHz, which is consistent with the frequency of intersection between the beam line and TM_01_ mode in Fig. 2(a). Because the frequency spectrum is very pure, the operation mode of the device in the SWS should be TM_01_ mode. Figure 3(d) enumerates the radial distributions of *E*_*z*_ within three different period positions of the SWS. Obviously, the electric field indeed takes its maximum value near the inner surface of the SWS, and all the three distributions agree well with the theoretical prediction of TM_01_ mode surface wave. This further confirms the fundamental mode operation in this oversized SWO. Besides, there is no envelope fluctuation for *E*_*z*_, and the relative half-width of the frequency spectrum is less than 0.5%, so no axial mode competition turns up in this device either. The average output power determined by integration of the Poynting flux across the output waveguide is shown in Fig. 3(e), where the output power from the device with PEC material is also illustrated for comparison. Due to the ohmic loss of copper, the output power is about 44.7 MW and the corresponding efficiency is about 5.4%, almost half of the ideal case. So the impact of metal conductivity on the output power becomes much more serious than the devices at subterahertz frequency band^39^.

## Experimental Setup and Measurement Results

**Figure 4:**
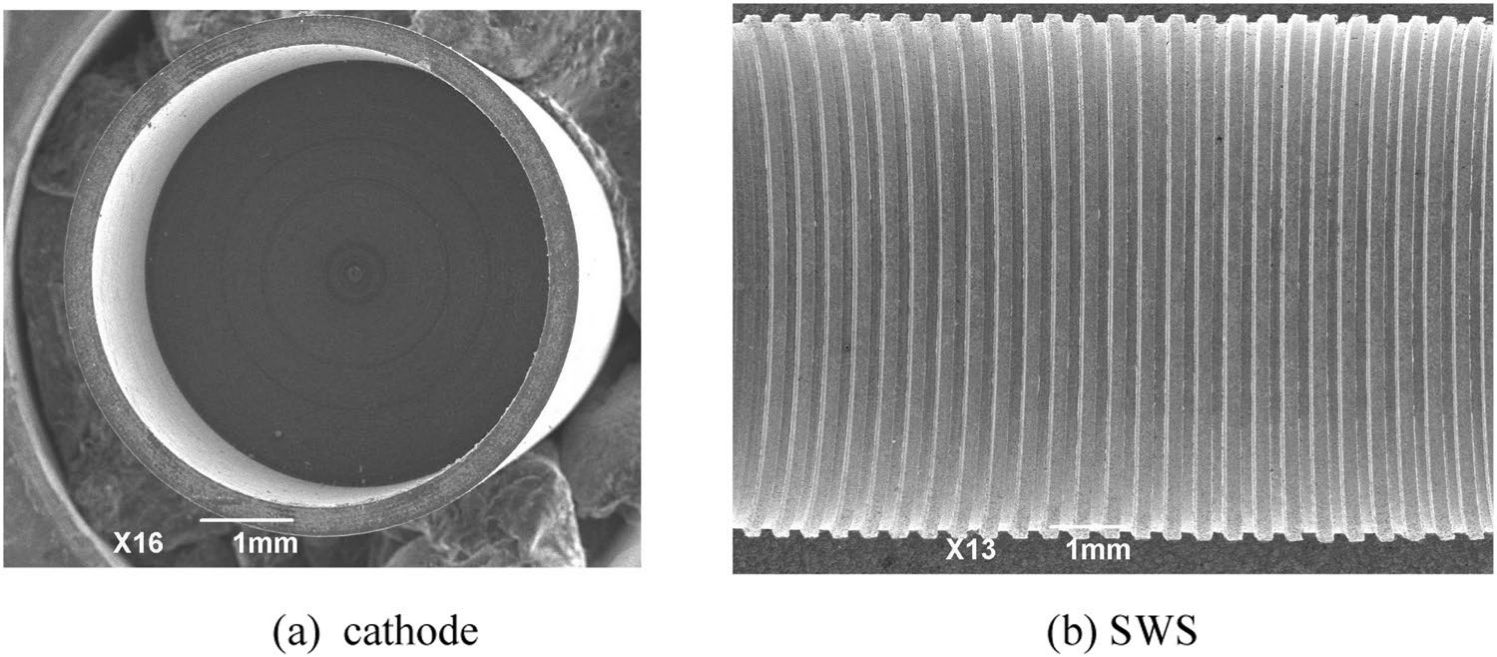
SEM images of cathode and SWS.

The experimental setup of the oversized SWO is shown in Fig. 1, including a compact accelerator, a solenoid coiled directly on the outer wall of the tube, the foilless diode, SWS inside, a radiation horn, and a vaccum system. The overall dimensions of the device are less than 3 m (length) ×1 m (width) ×1 m (height), satisfying the vehicle needs for future applications. The CKP3000 accelerator provides high voltage pulse with duration of about 8 ns on the foilless diode. The guiding magnetic field of 3.1 T is measured inside the solenoid by a Tesla meter. The explosive emission cathode is made of modified graphite as shown in Fig. 4(a). Measured results by a scanning electron microscope (SEM) indicate that its fabrication errors are less than 3.5%. This type of graphite cathode has been successfully used in the 0.14 THz repetitive SWO with life time of about fifty thousands of shots^27^. The oxygen-free copper SWS is manufactured by computer numerical control machining technique, and the inside SEM imaging is shown in Fig. 4(b). Dimension tolerances of the period length and depth are within 5% while the width’s is less than 10% according to the further results of a three dimensional profile scanning inspection.

**Figure 5:**
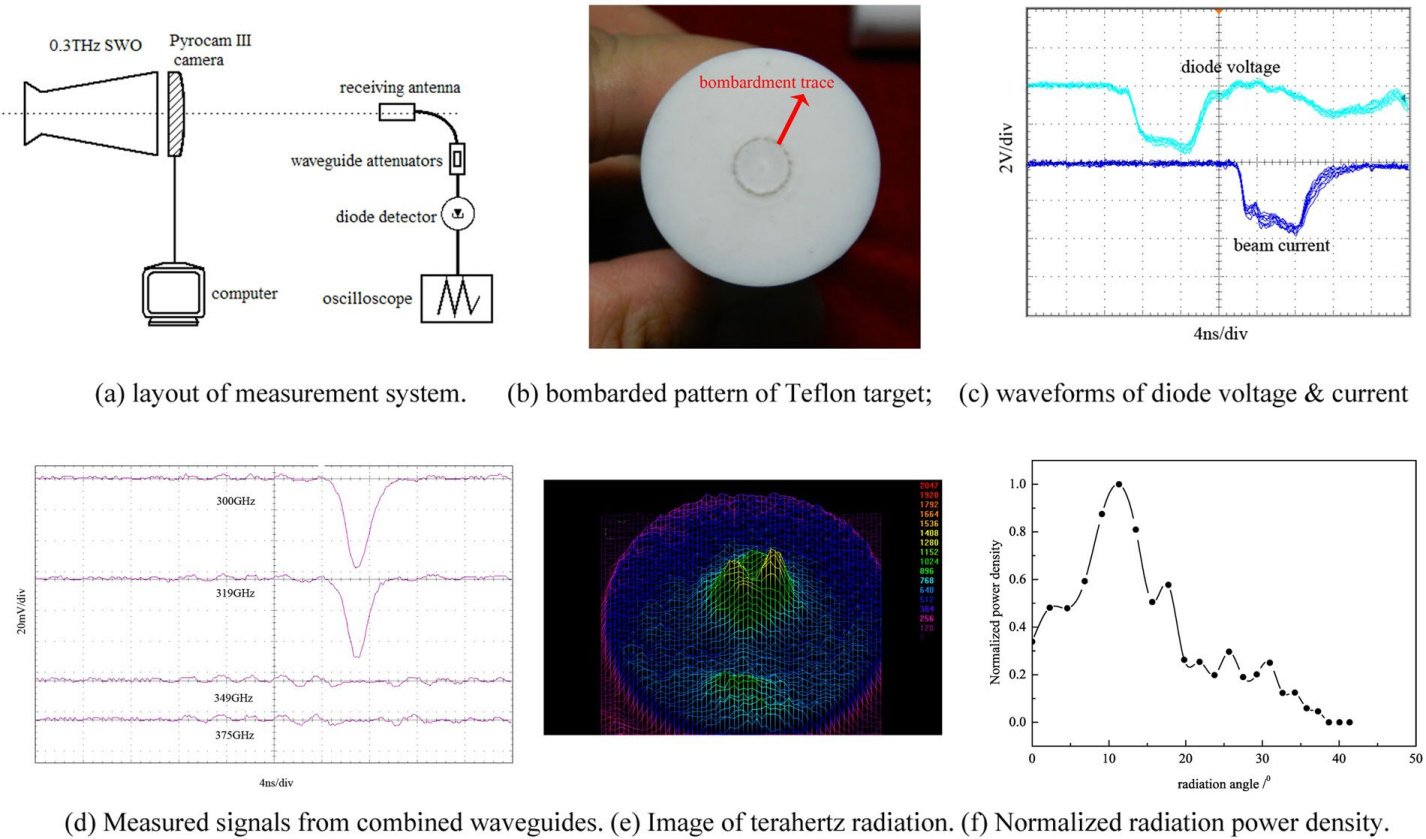
Experimental layout and results.

As the distance from the intense electron beam to the tube wall is merely 0.3 mm, excellent concentricity of the beam in the device should be achieved to ensure effective beam-wave interaction and try avoiding the generation of asymmetric modes. The diagnoses of beam position in radial direction are made by using a movable Teflon stick to fully intercept the beam. By judging whether these bombardment traces on the target at different distances overlap with the anticipated circle, we find an acceptable experimental state after dozens of tests and adjustments. Figure 5(b) shows the ultimate damage patterns of electron beam on Teflon target. Reasonable axial collimation is observed, and there are no remarkable absence and enhancement of electron beam in azimuthal direction. Then, the diode voltage and beam current are measured by a capacitive voltage divider and a Faraday cup, respectively. The typical measured results of ten shots are shown in Fig. 5(c). According to the calibration results of the divider and cup, the beam voltage is estimated to range from 350 kV to 480 kV with current in range of 2.3 ~ 3.6 kA by tuning the breakdown voltage of the gas switch of accelerator, satisfying our design requirements.

The terahertz pulses are diagnosed in the radiation field of the experimental setup, and the layout of the measurement system is schematically illustrated in Fig. 5(a). The pulse detecting system, composed of receiving antenna, waveguide attenuators, and diode detector, is used to determine the operation frequency range of the SWO. Taking different open-ended rectangular waveguides with cut-off frequencies of 300, 319, 349, and 375 GHz as the receiving antennas, we obtain the pulse frequency range by whether there is output from the detector. Four typical measured waveforms from the nominal high-pass filtering system are combined in Fig. 5(d). As the lengths of these open-ended waveguides are all 5 cm, that is, the attenuation for the frequency components below cut-off frequency is enough, the estimated operation frequency of the experimental device is in the range of 0.319–0.349 THz, which is consistent with the theoretical prediction in Fig. 2(a) and simulated result in Fig. 3(c). The frequency measurement results attest this device is indeed operated in TM_01_ mode. Besides, the terahertz pulse duration is about 2 ns as shown in Fig. 5(d).

Before the radiation power measurement, we diagnose the power distribution pattern near the radiation horn by using the Pyrocam III camera, which has a frequency response from 0.1 to 28.3 THz and an imaging area of 12.4 mm × 12.4 mm. As shown in Fig. 5(a), the pyroelectric camera is installed at a distance of about 2 mm from the horn with radius of 6 mm. Typical image in the active area is displayed in Fig. 5(e). Obviously, the radiation power distribution of the device indicates fairly good circular symmetry, and there is a distinct energy ring near the center. A small area of energy peak is also found below the energy ring, but its amplitude is much smaller. No breakdown marks are found on the radiation horn and SWS after experiments.

Based on the reasonable circular symmetry of the radiation field, the radiated pulse power is estimated using the pulse detecting system by integrating the power density over the radiation pattern. Figure 5(f) shows the normalized power density distribution measured in a quarter of spatial radition field in the horizontal direction. The density peak occurs at the angle of about 11^0^, and then it decreases rapidly towards the center and outside. Moreover, the dominated power localizes within the angle of about 40^0^. Except the distribution near the radiation edge, the measured power density distribution agrees with the near-field radiation pattern in Fig. 5(e). When the detecting system is calibrated by using a backward wave oscillator with operation frequencies ranging from 0.27–0.38 THz, the radiation power is evaluated to be about 2.1 MW. Compared with above PIC simulation prediction, there is a considerable disparity in the output power. In fact, the power disparities widely exist in the developments of high frequency VEDs as reported^3,27,28,33,40^, since the actualization of high quality electron beam with small size, perfect assembly and fabrication of SWS, and other issues are challenging. Besides, the short duration of the high voltage pulse from the accelerator is either an important cause. As seen from Fig. 3(e), the ohmic loss of the metal wall almost doubles the startup time of the device. So the oscillation and amplification of the terahertz wave are not sufficient within the duration of the applied high voltage, leading to the power decrease. In the actual experiments, the surface roughness of the tube, which becomes comparable to the skin depth, would introduce additional loss and deteriorate this situation^39^. Thus increasing the duration of the applied voltage pulse by upgrading the compact accelerator is necessary for the performance improvements and future applications of the proposed terahertz oversized SWO.

## Conclusions and Discussions

The design and experiments of a megawatt-level Y-band oversized SWO are presented. The cylindrical device driven by annular relativistic electron beam is featured by its compactness and high oversized ratio of about 6.8. By choosing appropriate beam-wave interaction point on the dispersion curve and beam position, we take advantage of the surface wave of TM_01_ mode to make sure that only TM_01_ mode can be excited while higher TM_0*n*_ modes are well suppressed. This is approved by the PIC simulation results. Based on a compact accelerator and the solenoid magnet, experimental setup is built to demonstrate its actual performance. Experimental results show that, the terahertz pulse is generated with a peak power of about 2.1 MW, a certain frequency in the range of 0.319–0.349 THz, and duration of about 2 ns. The radiation frequency coincides with our theoretical prediction and PIC simulation result, proving the feasibility of fundamental mode operation of the SWO with highly oversized ratio. However, the output power does not accord with the expectation since the electron beam quality, fabrication and assembly tolerances, and duration of the applied high voltage pulse still need further improvements. Next, we will further optimize the parameters of the structure and the driving electron beam^41^. Meanwhile, we will try to speed up the start time of the terahertz signal by injecting the external small terahertz signal^42^ with our newly developed continuous-wave Y-band planar BWO^43^.
